# Use of Pilocarpinum Muriaticum in Children’s Diseases

**Published:** 1879-09

**Authors:** 


USE OF PILOCARPINUM MURIATICUM IN CHILDREN'S DISEASES.—Prof. Weiss has studied the effects of pilocarpine in fourteen children suffering from nephritis, with general dropsy, following scarlatina. In four of the cases there existed, also, extensive bronchitis, in two diphtheria, and in one pneumonia of the left lung. The medicine was administered hypodermically, four or five drops of ether being added where collapse was feared. The following are the author's conclusions : 1. Pilocarpine has proved to be a very successful remedy for children suffering from nephritis and scarlatina. 2. In giving it to children, care should be taken to begin at first with small doses, which may later on be gradually increased. 3. If the little patients are very weak and likely to collapse after the injection, a few drops of ether should be added to the pilocarpine solution. 4. The drug produces a very copious and lasting secretion of sweat, such as no other drug has ever been known to call forth. It acts quickly. 5. In cases of bronchitis, complicated by dropsy, which often produces dyspnœa in children, the affection of the bronchi vanishes very soon after the remedy has been administered.—*London Medical Record.*

---

TO CLEANSE BOTTLES.—Dissolve one ounce of chloride of lime in one quart of water, and fill the bottles with the liquid; set them aside for several days and rinse them well with water. The water of chloride of lime can be used several times. For bottles which are not very dirty, use one part of muriatic acid diluted with three parts of water. Sawdust put into bottles and some water added, will clean well, especially such bottles as have contain ed oil.—*Pharm Handelsbl.*

